# Targeted Next-Generation Sequencing of *MLH1, MSH2*, and *MSH6* Genes in Patients with Endometrial Carcinoma under 50 Years of Age

**DOI:** 10.4274/balkanmedj.2018.0922

**Published:** 2019-01-01

**Authors:** Taha Reşid Özdemir, Murat Alan, Muzaffer Sancı, Altuğ Koç

**Affiliations:** 1 Genetic Diagnostic Center, Health Sciences University, İzmir Tepecik Training and Research Hospital, İzmir, Turkey; 2 Clinic of Gynecology and Gynecologic Oncology, Health Sciences University, İzmir Tepecik Training and Research Hospital, İzmir, Turkey

**Keywords:** Endometrial carcinomas, Lynch syndrome, mismatch repair, sequence analysis

## Abstract

**Background:**

Lynch syndrome is an inherited cancer disorder that causes an increased lifetime risk of various types of cancers. Endometrial cancer is the most common extracolonic cancer in Lynch syndrome. Guidelines recommend that patients with endometrial cancer younger than 50 years of age should be evaluated for Lynch syndrome. Molecular analysis of the mismatch repair genes and *EPCAM* gene is required for a definitive diagnosis of Lynch syndrome.

**Aims:**

To report the mutation analysis of mismatch repair genes using targeted next-generation sequencing in endometrial cancer diagnosed patients <50 years of age.

**Study Design:**

Retrospective cross-sectional study.

**Methods:**

Seventy-nine endometrial cancer diagnosed patients <50 years of age underwent genetic counseling. They were selected among 1094 consecutive endometrial cancer patients between 2006 and 2017. Molecular analysis of *MLH1, MSH2*, and *MSH6* genes was performed in 79 patients by using next-generation sequencing. Deletion/duplication analysis of mismatch repair genes and EPCAM gene was also performed in 79 patients by using the multiplex ligation-dependent probe amplification method.

**Results:**

Germline testing of mismatch repair genes was performed in 79 endometrial cancer patients. Lynch syndrome was confirmed in 4 patients (5%; 4/79). A total of 14 variants (6 in *MSH2*, 5 in *MLH1*, 3 in *MSH6* genes) were found in 14 patients. Four variants were assessed as pathogenic/likely pathogenic, and 10 variants were assessed as variants of uncertain significance.

**Conclusion:**

Lynch syndrome should be investigated in patients diagnosed with endometrial cancer that are less than 50 years of age due to the increased lifetime risk of developing cancer.

Lynch syndrome is an autosomal dominant disorder that causes colorectal cancer, endometrial cancer, and upper gastrointestinal tract cancers among others. Lynch syndrome occurs when there is a germline mutation in *MLH1*, *MSH2*, *MSH6*, *PMS2*, [mismatch repair (MMR) genes] and the *EPCAM* gene. The frequency of mutation carriers in MMR genes is estimated to be 1:279 (1).

There are several criteria to identify individuals at risk for Lynch syndrome. These include clinical and pathologic criteria (e.g. Amsterdam criteria, revised Bethesda criteria), tumor testing [microsatellite instability, immunohistochemistry (IHC) testing], and prediction models (PREMM_5_ model, MMRpredict model, MMRpro model).

Endometrial cancer is the most common extracolonic cancer in Lynch syndrome that accounts for 2 to 5 % of all endometrial cancers (2,3). The women with Lynch syndrome have an increased cancer risk during their lifetime for endometrial cancer (25-60%), colorectal cancer (40-80%), and ovarian cancer (4-24%) (4). According to the American College of Medical Genetics and Genomics (ACMG) and the National Society of Genetic Counselors (NSGC) guideline (4), patients diagnosed with endometrial cancer younger than 50 years of age should be evaluated for Lynch syndrome. In these patients, performing a germline testing in MMR genes confirms the diagnosis and it could guide the clinical treatment. In addition, it could provide a mutation screening for asymptomatic relatives who will be at 50% risk of inheriting the mutation.

In our study, the molecular analysis of MMR genes was performed using targeted next-generation sequencing (NGS). This provides simultaneous analysis of multiple genes in a single test, in patients diagnosed with endometrial cancer younger than 50 years of age, to present the frequency of germline mutations in MMR genes. To our knowledge, this is the first study of this kind in Turkey.

## MATERIALS AND METHODS

### Patients

The ethical committee approved this study as a retrospective study, and informed consent was obtained from the patients studied. The gynecologic-oncology department reviewed the files of patients with endometrial cancer between the years 2006-2017. They identified 79 patients diagnosed with endometrial cancer younger than 50 years of age among 1094 patients. Seventy-nine patients were directed to the genetic diagnostic center for genetic counseling. When the patients’ records were examined, it was noticed that the IHC or microsatellite instability testing was not requested. After the family history was reviewed for each patient, the PREMM_5_ prediction model was used to predict the probability of a patient carrying a germline mutation in the MMR genes, or *EPCAM* gene (5).

### Targeted NGS

After genomic DNA samples were obtained, targeted NGS was performed with the Illumina MiSeq NGS System (Illumina Inc., San Diego, CA, USA) using a Miseq Reagent Kit v2 (500-cycles) (Catalog No: MS-102-2003. Illumina Inc., San Diego, CA, USA). The NEXTflex^®^ Colorectal Cancer Amplicon Panels for the Illumina^®^ platforms (Bioo Scientific Corp., Austin, TX, USA) were used to reveal variants in the coding regions and in the intronic regions (up to the area covered by the kit) of *MLH1*, *MSH2*, and *MSH6* genes. Analysis of the *PMS2* gene was excluded from the study due to a large number of pseudogenes belonging to this gene.

### NGS data analysis

The raw data obtained with the NGS method was analyzed using the ‘SEQ variant analysis software’ (Genomize, İstanbul, Turkey) according to the reference genome of GRCh37 (hg19) [RefSeqID’s: *MLH1* (NM_000249), *MSH2* (NM_000251), *MSH6* (NM_000179)]. The SEQ software demonstrated that the minimum coverage-depth of the target regions was 100X. These were evaluated using the Integrative Genomics Viewer software (6,7). Variants were determined based on 50X coverage-depth per allele (reference allele/alternative allele) and they were filtered according to following criteria:

• Exclusion of benign (B)/likely benign variants from all the submissions in the ClinVar database,

• Exclusion of variants that had an allele frequency >5% in any of the population databases (ESP or 1000 Genomes or ExAC),

• The inclusion of variants in the coding regions and in the intronic regions.

Lastly, filtered variants were evaluated according to the ACMG Standards and Guidelines recommendations (8). Several databases and in-silico prediction tools were used for interpreting these variants (9,10,11,12,13).

### Confirmation and Multiplex ligation-dependent probe amplification analyses

The ACMG Standards and Guidelines (8) recommend performing confirmation studies for all sequence variants that are considered to be pathogenic or likely pathogenic. Therefore, Sanger sequencing was performed in patients with variants that were considered to be pathogenic or likely pathogenic. After NGS analysis, multiplex ligation-dependent probe amplification (MLPA) analysis was performed using SALSA^®^ MLPA^®^ probemix P003-D1 *MLH1*/*MSH2*, P072-C1 MSH6 kits (MRC-Holland, Amsterdam, The Netherlands) in patients who had no pathogenic or likely pathogenic variants.

## RESULTS

Molecular analysis of MMR genes was performed in 79 patients with endometrial cancer. The mean age at diagnosis for all the patients was 44.4 years, with a range of 22 to 49 years. The mean age at diagnosis for four patients with pathogenic/likely pathogenic variants was 42.5 (range 35 to 48 years). Family history and pathologic features of patients with identified variants are presented in Table 1.

Fourteen different variants were identified in 14 patients (18%; 14/79) (Table 2). Four variants were assessed as pathogenic or likely pathogenic that were confirmed using Sanger sequencing. Thus, Lynch syndrome diagnosis was confirmed in 4 patients (5%; 4/79). Ten variants were assessed as ‘variants of uncertain significance’. All of the variants were found to be heterozygous. Three variants were interpreted as pathogenic and one was interpreted as likely pathogenic (5%; 4/79). One of the pathogenic variants (in the *MLH1* gene) was previously reported in the literature (14,15). The others were evaluated as a novel (1 in *MLH1* and 2 in *MSH2*). Four variants in *MSH2*, 3 variants in *MLH1*, and 3 variants in *MSH6* genes were found and evaluated as variants of uncertain significance.

No deletion or duplication was detected in *MLH1*, *MSH2*, *MSH6* genes, and *EPCAM* gene.

## DISCUSSION

Several criteria, such as the Amsterdam and Bethesda criteria, are used to identify cases at risk for Lynch syndrome in endometrial cancer patients; however, not all the patients with Lynch syndrome meet these criteria. The mean age at diagnosis is between 46 and 54 years in endometrial cancer patients with Lynch syndrome (16). In addition, a significant proportion (10%) of endometrial cancer patients with Lynch syndrome is diagnosed at an age younger than 50 years of age (17). Therefore, patients with endometrial cancer diagnosed less than 50 years of age were evaluated for Lynch syndrome in our study and the ACMG and NSGC guideline were considered. For this purposes, we performed molecular analysis of MMR genes (*MLH1*, *MSH2*, and *MSH6* genes) by using targeted NGS that provides a simultaneous analysis of genes at a comparable cost to Sanger sequencing.

**Figure 1:**
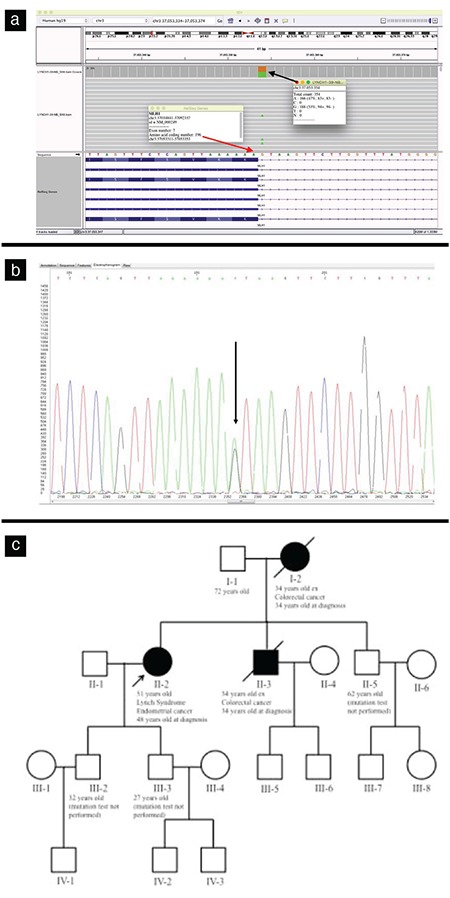
IGV image (a), electropherogram (b) pedigree (c) patient no: 46 with a splice site variant [MLH1:NM_000249:c.588+1G>A(Intron7) Heterozygous]. The black arrow indicates nucleotide change “+1G>A”. The red arrow indicates the last nucleotide of the exon7 of *MLH1* gene. IGV: integrative genomics viewer

Two novel variants in the *MSH2* gene were identified in this study. The first novel variant was an inframe deletion that was found between 416-418 nucleotides position. There was a known disease mutation at the 416. nucleotide position [c.416delA(p.N139Mfs*35)](HGMD:CD056196), three nucleotide deletions were found in our study. The second was a frameshift variant consisting of five nucleotides deletion that affected the 744. amino acid position. There was a mutation consisting of a single nucleotide change in the same codon that was previously reported [c.2231T>G(p.L744*)](HGMD: CM117434). One novel variant in *MLH1* gene was found in the donor splice site of the intron7 (NM_000249:c.588+1G>A) (Figure 1a, 1b). There was a pathogenic variant previously established in the same position [NM_000249:c.588+1G>T(HGMD: CS065593)] but substitutions were different. The patients who had a pathogenic variant in *MSH2* gene did not have a positive family history, whereas the patients in whom pathogenic variants were found in *MLH1* gene did have a positive family history (Figure 1c).

Several studies were reported in the literature (Table 3); however, we could not find any NGS or Sanger sequencing studies in patients with endometrial cancer associated to Lynch syndrome in the Turkish population to compare with our study. Berends et al. (18) performed a mutation analysis of the *MLH1*, *MSH2*, and *MSH6* genes using Sanger sequencing and MLPA techniques in 57 patients with endometrial cancer, who were younger than 50 years of age. They found 5 pathogenic germline mutations (1 in *MLH1*, 3 in *MSH2*, and 1 in *MSH6*) in 57 patients (8.8%; 5/57). In our study, we found 4 pathogenic variants in 79 patients (5%; 4/79). In another study, Goodfellow et al. (19) investigated only the *MSH6* gene using sequencing. They performed microsatellite instability and *MLH1* methylation analyses in 441 endometrial cancer patients with no personal or family cancer history. They evaluated 100 cases (23%; 100/441) for the *MSH6* gene and found 7 germline mutations. Finally, they estimated the minimum prevalence of the *MSH6* mutation was 1.6% (7/441). We did not find any pathogenic variant in the *MSH6* gene in our study. One of the reasons could be that all patients diagnosed were <50 years. Several studies have shown that endometrial cancer patients with the *MSH6* germline mutations are associated with an older age (typically above 50 years) compared with *MLH1* and *MSH2* mutation carriers (19). The other study was performed by Ollikainen et al. (20). They screened *MLH1*, *MSH2*, *MSH6* genes, and MLPA by Sanger sequencing in 32 cases among 519 consecutive patients with endometrial cancer. They found 11 mutations (6 in *MLH1*, 4 in *MSH2*, 1 in *MSH6* genes). The minimum incidence of the MMR gene germline mutations was 2.1% (11/519) while in their study they found 0.3% (4 of 1094). One of the reasons could be that the *PMS2* gene was not analyzed in this study. Hampel et al. (16) performed a large study that consisted of 543 endometrial cancer patients. They performed microsatellite instability testing in 543 tumors. One hundred eighteen (21.7%) were microsatellite instability positive. They used sequencing and MLPA methods for analysis of the *MLH1*, *MSH2* and *MSH6* genes in 118 patients (MSI+). Ten mutations were identified (1 in *MLH1*, 3 in *MSH2*, 6 in *MSH6* genes). The mean age of these patients was 54.6. They emphasized that at least 1.8% (10/543) of all endometrial cancer patients had Lynch syndrome. Four of the 81 patients (4.9%; 4/81) who were diagnosed under age 50 had Lynch syndrome, this was nearly identical to our findings (5%; 4/79). Lu et al. (21) performed a study consisting of 100 endometrial cancer patients diagnosed <50 years. *MLH1*, *MSH2*, and *MSH6* genes were analyzed by sequencing and MLPA. They found 9 germline mutations (1 in *MLH1*, 7 in *MSH2*, 1 in *MSH6* genes). The mean age at diagnosis was 41.6 years. In another study by Anagnostopoulos et al. (22), MMR germline mutation test was performed and they identified 3 pathogenic MMR mutations in 3 of 35 patients with endometrial cancer under age 50 (8.5%; 3/35).

Lynch syndrome patients and their relatives who have the same mutation can benefit from surveillance programs that could improve the chances of earlier diagnoses and reduce cancer risks (23,24,25,26). Prophylactic hysterectomy and bilateral salpingo-oophorectomy are recommended in patients with Lynch syndrome who have finished childbearing or have more than 40 years of age. Before taking this decision, patients should be evaluated by the gynecologic oncologist. Colonoscopy screening is also recommended in affected persons and their first-degree relatives.

In conclusion, endometrial cancer patients younger than 50 years of age should be evaluated for Lynch syndrome. Germline testing of MMR genes is required for definitive diagnosis of Lynch syndrome. This is the first Turkish study to present the experience of a single center in terms of revealing the mutation frequency of MMR genes using the NGS method in patients with endometrial cancer diagnosed <50 years. In the future, further studies are needed in larger groups.

## Figures and Tables

**Table 1:**
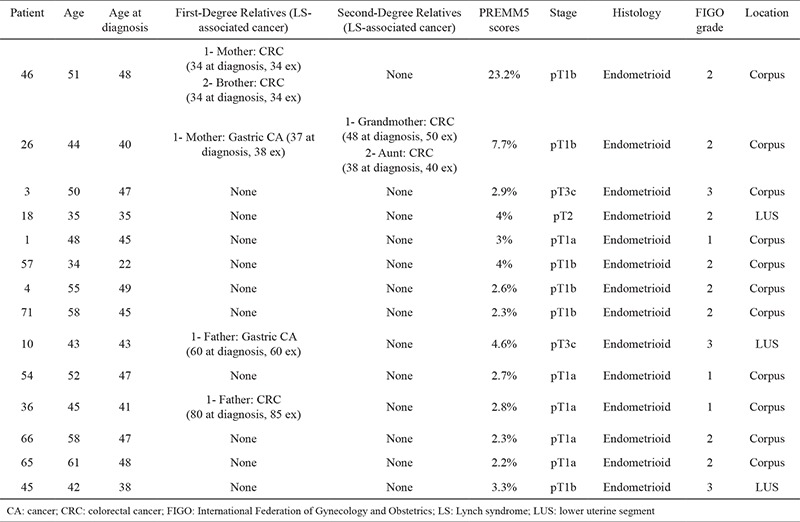
Summary of patients family history and pathologic features of patients with an identified variations (n=14)

**Table 2:**
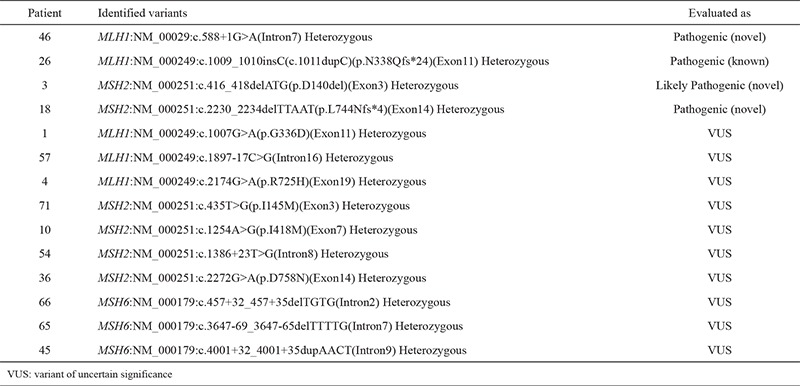
Classification of variants identified (n=14)

**Table 3:**
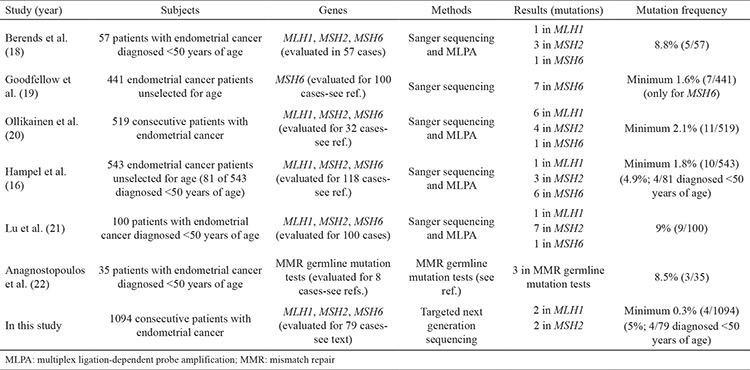
Summary of molecular analysis of MMR genes studies in patients with endometrial cancer
